# Air Embolism after Central Venous Catheter Removal: Fibrin Sheath as the Portal of Persistent Air Entry

**DOI:** 10.1155/2013/403243

**Published:** 2013-10-07

**Authors:** Meggiolaro Marco, Erik Roman-Pognuz, Baritussio Anna, Scatto Alessio

**Affiliations:** ^1^Division of Anaesthesiology and Intensive Care, University Hospital of Padova, Via Giustiniani 2, 35128 Padova, Italy; ^2^Division of Cardiology, University Hospital of Padova, Via Giustiniani 2, 35128 Padova, Italy

## Abstract

Central venous catheterization is of common practice in intensive care units; despite representing an essential device in various clinical circumstances, it represents a source of complications, sometimes even fatal, related to its management. We report the removal of a central venous catheter (CVC) that had been wrongly positioned through left internal jugular vein. The vein presented complete thrombosis at vascular ultrasonography. An echocardiogram performed 24 hours after CVC removal showed the presence, apparently unjustified, of microbubbles in right chambers of the heart. A neck-thorax CT scan showed the presence of air bubbles within the left internal jugular vein, left innominate vein, and left subclavian vein. A vascular ultrasonography, focused on venous catheter insertion site, disclosed the presence of a vein-to-dermis fistula, as portal of air entry. Only after air occlusive dressing, we documented echographic disappearance of air bubbles within the right cardiac cavity. This report emphasizes possible air entry even many hours after CVC removal, making it mandatory to perform 24–72-hour air occlusive dressing or, when inadequate, to perform a purse string.

## 1. Introduction

Venous air embolism is a well-known complication of venous catheterization in critically ill patients: it is generally related to insertion and removal procedures and daily management.

Late air embolism after CVC removal is less known. We describe the case of a nonlethal air embolism 24 hours after removal of a malpositioned CVC, placed through left internal jugular vein, which was completely occluded by a thrombus.

## 2. Case Presentation

A 75-year-old woman was admitted to our intensive care unit for clinical monitoring after right parotid gland removal; she was diagnosed with a colliquative tumoral parotid gland mass in a peripheric hospital and then moved to the local otolaryngological surgical department to undergo surgery.

Her past history included hypertensive cardiomiopathy, with episodes of heart failure, and atrial fibrillation.

**Figure 1:**
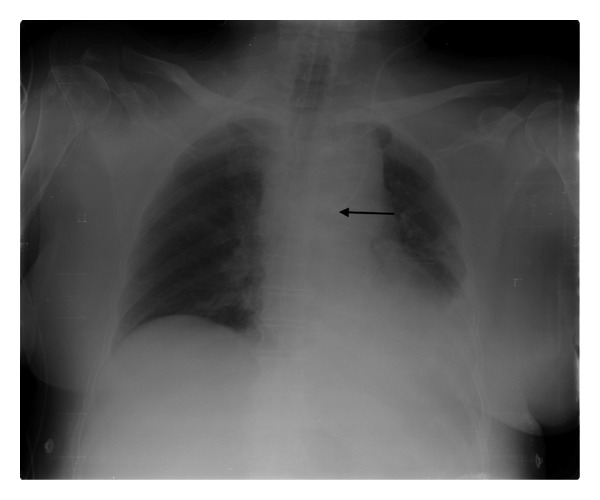
Chest radiograph showing tip (arrow) projecting over the left side of the descending aorta, at the level of carina, creating an angle with the spine greater than 40°.

On admission to our unit she underwent a chest radiograph that showed a wrong positioning of the CVC (arrow 7 Fr, 3 lumen, and 16 cm length): as shown in Figure 1 the tip projected over the left side of the descending aorta, at the level of carina, creating an angle with the spine greater than 40° [1]. Moreover, we noticed saline leak from the insertion site and suspected a catheter rupture. In addition we performed a vascular ultrasonography that showed a complete thrombotic occlusion of the left internal jugular vein.

For these reasons and for the timing of catheterization (28 days before) we decided to remove it.

The patient was mechanically ventilated with assisted mode, and she was placed in the head-down position before removing the catheter. Resistance was met during the removal of the catheter; after its dislodgement, local pressure using a gauze was performed to avoid bleeding. At inspection the catheter was whole, and a brownish scab on its distal tip compatible with a blood clot was found.

Afterwards she developed hemodynamic instability (blood pressure 86/40 mmHg, heart rate 100 bpm) that required adding inotropic support (dopamine 6 mcg/Kg/min) and deterioration of ventilation (PaO_2_/FiO_2_ 146).

**Figure 2:**
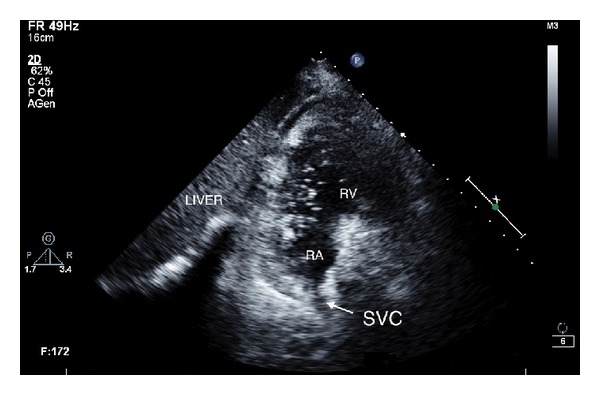
Echocardiography. Subcostal acoustic window: air bubbles in right atrial and ventricular cavities. RA: right atrium; RV: right ventricle; SVC: superior vena cava.

Due to no evidence of recovery, the next day we performed an echocardiogram and noticed air microbubbles in the right heart chambers (Figure 2); these were synchronous with breaths and were apparently coming from superior vena cava. On the hypothesis of a vein-to-dermis fistula made while removing the catheter, the insertion site was sealed with air occlusive dressing with no more evidence of air bubbles within the heart.

**Figure 3:**
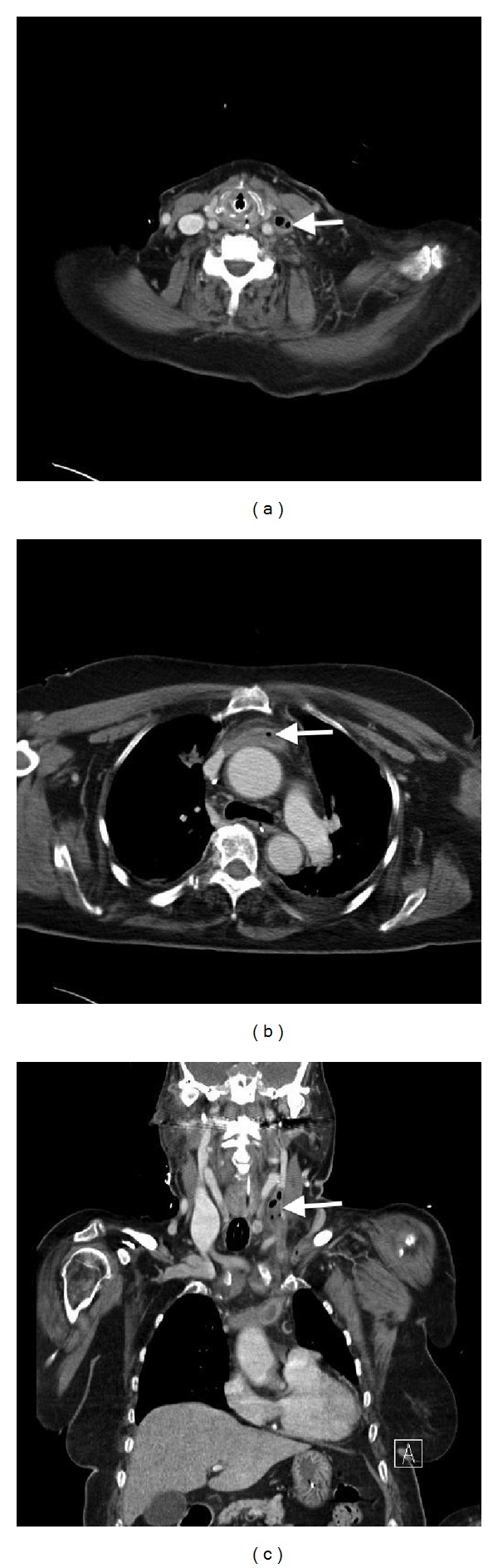
Neck and thoracic tomodensitometry in ((a), (b)) coronal and (c) frontal planes showing total thrombotic occlusion of the left internal jugular vein extending to the innominate vein and partially involving the left subclavian vein, with air bubbles within it (arrows).

A neck and thorax CT scan was performed, showing total thrombotic occlusion of the left internal jugular vein extending to the innominate vein and partially involving the left subclavian vein, with air bubbles within it (Figure 3). A diagnosis of venous air embolism associated with removal of central venous catheter was then made.

**Figure 4:**
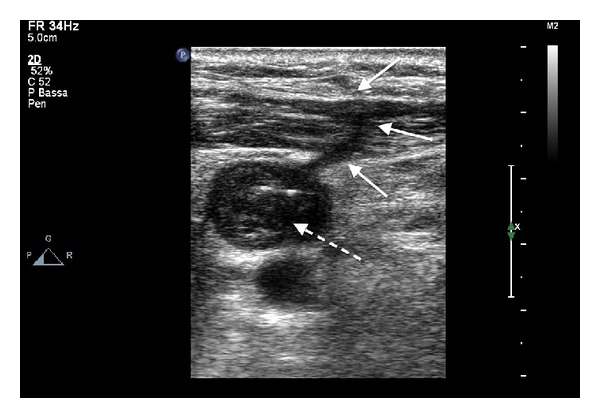
Vascular ultrasonography showing total thrombotic occlusion of the left internal jugular vein (dotted arrow) and fibrin sheath between the vein and the dermis (white arrow).

At a second vascular ultrasonography we noticed the presence of a tract between the vein and the dermis (Figure 4), responsible for air embolism, which was still visible more than 24 hours after air occlusive dressing placement.

## 3. Discussion

Central venous catheter removal is an awkward and sometimes potentially fatal procedure that requires as much practice as its placement [2].

Serious complications account for less than 1% of all catheter insertions, but mortality may reach 50% [3].

Review of the literature identifies many cases of air embolism occurred after catheter removal, determining occlusion of pulmonary vasculature [3–7], cerebral vessels [8], and coronary arteries [9, 10].

Even though generally related to inappropriate removing technique, venous air embolism may be caused by persistence of a subcutaneous tract [3, 4, 6, 8, 11].

Fibrin tracts may be formed around the catheter, especially after a long stay, but sometimes even within 24 hours [4, 6], creating a portal of air entry. These tracts usually lyse spontaneously but may predispose to thrombus formation [3].

Risk of venous air embolism is increased by conditions that decrease central venous pressure, such as deep inspiration, hypovolemia, and upright position during removal [11]; even coughing might be dangerous because it might dislodge a thrombus plug [7] or separate soft tissue around the catheter [11].

Mennim et al. [6] and Boer and Hene [11] suggested some recommendations to avoid venous air embolism after catheter removal: no heparin should be administered when catheter removal is foreseen, or protamine should be given in cases of heparin administration prior to removal; the patient should be in the head-down position, so that the exit site is below the level of the right atrium and should be aware not to cough, talk, or inspire deeply during the procedure.

Exit site should be firmly pressed with gauze until haemostasis is achieved and then sealed with an air occlusive dressing; there is no agreement on definitive length of time for dressing keeping, but 24–72 hours might be advisable [6].

We report the case of air embolism through documented fibrin tract, mainly due to long-standing catheter and complicated by thrombus formation. Surprisingly, fibrin tract was visible even more than 24 hours after catheter removal, and this supports the general idea to leave air occlusive dressing longer, at least 24–72 hours.

Our patient was mechanically ventilated which should have avoided decreasing pressure in venous system; by the way, since she was conscious and on pressure support, we presume that her inspiratory trigger was enough to make venous pressure subatmospheric.

Because of the sometimes catastrophic consequences of air embolism, all recommendations on catheter removal should be pursued and followed. More attention should be dedicated to exit site sealing and perhaps tighter dressing. Performing a purse string would be advisable in certain subgroups of patients as the elderly ones, those with frail subcutaneous tissue and with delayed healing condition (diabetes, malnutrition, and steroid therapy).
